# MRI‐Based Assessment of Microfocused Ultrasound‐Induced Midface Lifting

**DOI:** 10.1111/jocd.70477

**Published:** 2025-10-21

**Authors:** Jin Ho Kim, Won Seok Choi

**Affiliations:** ^1^ V&Co Cooperation Seoul Korea; ^2^ V‐Plastic Surgery Daegu Korea

**Keywords:** facial lifting, microfocused ultrasound, subcutaneous fat, superficial musculoaponeurotic system

To the Editor,

1

Microfocused ultrasound (MFU) generates thermal coagulation zones, stimulating collagen production to promote skin regeneration. By targeting retinacula cutis (RC) within subcutaneous fat and the superficial musculoaponeurotic system (SMAS), MFU reinforces these structures, achieving noninvasive lifting [1]. While previous studies have demonstrated MFU effects through clinical photography and histopathological analysis, anatomical changes in the midface remain underexplored [1, 2]. Given its superiority in discriminating soft tissue and its ability to provide anatomical, quantitative data, magnetic resonance imaging (MRI) is the preferred modality for investigation [3].

A 32‐year‐old male seeking noninvasive midface lifting underwent pre‐treatment clinical photography and MRI scans in a supine position after providing informed consent. MFU treatment (Ultherapy Prime, Merz, North America) was administered under ultrasound guidance, delivering 240 shots with 0.9 J at 4.5 mm and 380 shots with 0.35 J at 3.0 mm to the midface. A follow‐up MRI was conducted 3 months post‐procedure, during which his BMI showed minimal change (23.0–23.3).

MRI measurements were performed at three points on the upper midface along a line connecting the nasal ala and tragus, with three sites of lower fat located 3.0 cm inferiorly (Figure 1A–C). Fat thickness, SMAS thickness, and μ values were assessed. Clinically, the Global Aesthetic Improvement Scale (GAIS) score was three, confirming obvious enhancement (Figure 2). Moreover, previous microscopic studies reported increased SMAS thickness in post‐MFU treatment [4]. Consistent with these findings, our study observed an approximate 0.1 mm increase in SMAS thickness (range from 0.03 to 0.16 mm) (Table 1).

**FIGURE 1:**
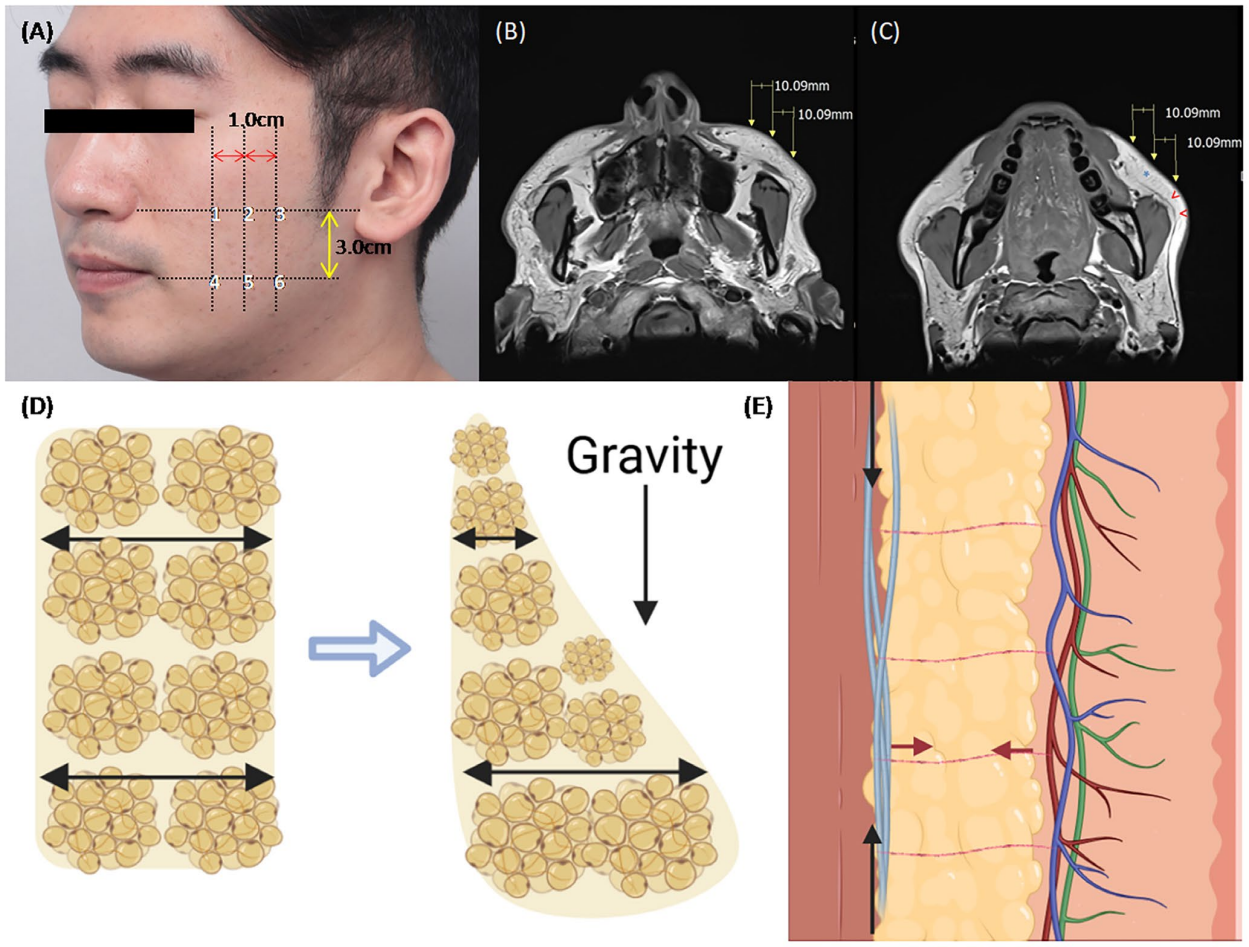
(A–C) 6 Points of measurements, red arrow head showed superficial musculoaponeurotic system and blue asterisk showed superficial fat layer with vertical fibrous septae on T1‐weighted MRI. (D) Superficial fat gliding to inferior forming droplet‐like shape in the sagittal plane. The ratio of fat thickness (upper over lower fat) become smaller with aging. (E) Superficial musculoaponeurotic system (SMAS) (blue line) contracts antigravital direction (black arrow) while fibrous septae (red line) contracts vertical to SMAS (red arrow).

**FIGURE 2:**
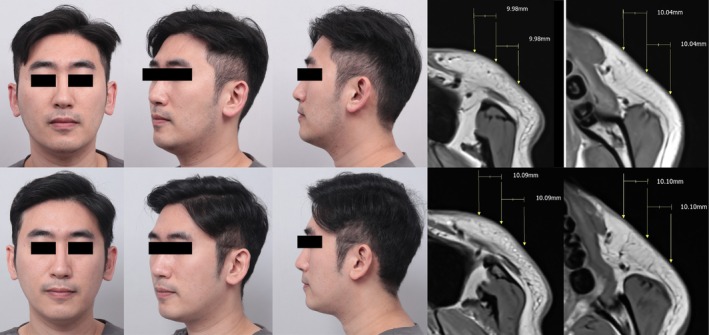
Clinical photograph and T1‐weighted MRI of pre‐treatment (upper) and post‐3 month (lower) with microfocused ultrasound.

**TABLE 1:** Result of superficial fat thickness, superficial musculoaponeurotic system thickness, fat thickness ratio (upper over lower), and calculated *μ* value of each 6 measuring sites.

		Upper‐medial	Lower‐medial	Upper‐middle	Lower‐middle	Upper‐lateral	Lower‐lateral
Fat thickness (mm)	Pre	6.68	8.33	10.08	8.19	6.18	4.84
	Post	5.69	6.71	8.52	6.01	6.31	3.87
SMAS thickness (mm)	Pre	0.9	0.6	0.94	0.73	0.77	0.80
	Post	0.93	0.72	1.00	0.81	0.88	0.96
Fat thickness ratio	Pre	0.76		1.23		1.28	
	Post	0.85		1.41		1.63	
*μ*	Pre	9.36		7.42		4.31	
	Post	7.31		5.12		3.12	

According to the fat‐gliding theory, facial aging results from laxity of the retinacula cutis (RC) and the superficial musculoaponeurotic system (SMAS), which support the superficial fat compartments [5, 6]. This laxity progresses inferiorly, displacing fat pads, yielding a droplet‐like shape in the sagittal plane (Figure 1D). With aging, the ratio of superior‐to‐inferior fat thickness declines (Figure 1D). In our study, we demonstrated a significant posttreatment increase in the superior‐to‐inferior fat thickness ratio (FTR) following MFU (Table 1). Thermal coagulation point (TCP) formed within the SMAS—aligned parallel to the skin—and the perpendicularly oriented RC likely produced tissue contraction and reinforcement, thereby driving both the lifting effect and the observed FTR changes (Figure 1E).

Although most treated areas exhibited reduced fat thickness, it remains unclear whether this decrease reflects RC tightening or lipolytic activity. Nonetheless, to mitigate the risk of lipoatrophy, especially in predisposed patients, real‐time ultrasound guidance is essential to avoid excessive TCZ formation within the RC layer. High‐resolution ultrasound studies have revealed marked variability in SMAS architecture across the midface, further highlighting the importance of precise imaging to ensure accurate thermal coagulation. Consequently, visualization‐based, target‐focused MFU‐V is paramount for precision treatment; blind application of MFU may inadvertently induce lipoatrophy or deposit TCZs at unintended depths, leading to suboptimal results and unanticipated complications.

Finally, we introduce a novel composite efficacy index—calculated as the product of FTR and mean fat thickness (Table 1)—to integrate the lifting effect conferred by SMAS contraction with the reduction in fat thickness. This metric offers a more holistic evaluation of MFU‐V efficacy. Future investigations involving larger cohorts are warranted to validate this index (Equation 1):

(1) $$\mu =\left(\frac{\text{lower thickness}}{\text{upper thickness}}\right)\times \left(\frac{\text{upper thickness}+\text{lower thickness}}{2}\right)$$

In summary, MRI analysis confirmed MFU‐induced anatomical changes in SMAS and subcutaneous fat thickness. Based on the fat‐gliding theory, an increase of FTR and a decrease of subcutaneous fat thickness showed anatomical change due to MFU leading to midface lifting and an anti‐aging effect. This is the first study about the radiologic change after MFU treatment. As a pilot study, this investigation is limited to a single case. Future research will expand upon these findings by analyzing a larger cohort to further validate and refine conclusions.

## Author Contributions

Dr. Jin Ho Kim contributed to data curation and analysis, investigation, and writing the original draft. Dr. Won Seok Choi contributed to conceptualization, validation, and reviewing the original draft.

## Consent

Participant provided written informed consent.

## Conflicts of Interest

The authors declare no conflicts of interest.

## Data Availability

The data that support the findings of this study are available from the corresponding author upon reasonable request.
